# Seroprevalence of equine leptospirosis in Poland (2019–2023)

**DOI:** 10.1111/evj.70069

**Published:** 2025-08-04

**Authors:** Jacek Żmudzki, Monika Ostrowska, Zbigniew Arent, Maciej Frant, Maciej Kochanowski, Agnieszka Nowak, Sylwia Zębek, Damian Kalinowski, Katarzyna Podgórska

**Affiliations:** ^1^ Department of Swine Diseases National Veterinary Research Institute Pulawy Poland; ^2^ Department of Infectious Diseases and Public Health Faculty of Veterinary Medicine, University of Agriculture in Krakow Krakow Poland

**Keywords:** horse, leptospirosis, MAT, serology

## Abstract

**Background:**

Leptospirosis in horses is associated with various clinical signs, potentially leading to fatal outcomes. Additionally, the disease may pose a zoonotic risk to individuals involved in handling infected animals. Implementing a serological monitoring programme in the equine population is one of the key tools used to reduce the risk of transmission of *Leptospira* infections to humans.

**Objectives:**

To provide new insights into the seroprevalence of leptospirosis in domestic horses in Poland.

**Study Design:**

Serological monitoring program.

**Methods:**

Data were collected from serological surveys of horse serum samples across 14 of the 16 provinces between 2019 and 2023. A total of 4474 horse serum samples were tested using the microscopic agglutination test (MAT), with 8 *Leptospira* serovars from 7 European serogroups. Statistical analyses and data visualisation were performed using Python‐based libraries. Prevalence was calculated as absolute and relative percentages. Pairwise prevalence comparisons employed chi‐squared tests with adjustments for multiple comparisons. Bayesian posterior probabilities were estimated to evaluate prevalence differences between groups.

**Results:**

The 5‐year study showed a relatively high exposure (25.1%) of horses to *Leptospira* antigens in Poland. Dominant *Leptospira* serogroups were: Sejroe (39.2%), Pomona (14.6%), Bratislava (11.2%), and Grippotyphosa (10.1%). Pomorskie recorded the highest percentage of positive samples (29.2%). Conversely, Lubuskie exhibited the lowest prevalence at 16.2%.

**Main Limitations:**

Details on the uses of horses were not available.

**Conclusions:**

This analysis provides valuable data on the circulation of *Leptospira* serogroups across different regions over time. The high seroprevalence of *Leptospira* in the Polish horse population underscores the necessity of ongoing monitoring, which will aid in the protection of individual horses, herds, and humans from potential infections.

## 1 INTRODUCTION

Leptospirosis is currently considered one of the most important zoonoses. Severe human leptospirosis is characterised by dysfunction of multiple organs including the liver, kidneys, lungs, and brain with typical clinical symptoms such as jaundice and renal failure.^1^ The mean case fatality ratio in human cases is estimated at the level of 6.9% (95% CI 5.66–8.03) worldwide.^2^ The disease occurs in many species of mammals including non‐human primates, rodents, domestic and farm animals, such as dogs, cats, pigs, cattle, small ruminants as well as in wildlife.^1^, ^3^, ^4^ In horses, clinical leptospirosis is primarily associated with neonatal death and embryonic absorption causing abortion in pregnant mares.^5^, ^6^, ^7^, ^8^, ^9^, ^10^, ^11^ Other clinical signs include lethargy, anorexia, fever, tachypnoea, abnormal lung sounds, epistaxis, and signs of uveitis leading in chronic cases to blindness. Kidney and liver dysfunction may occur with severe infections and can result in death.^8^, ^9^

According to the Polish Horse Breeders Association (PZHK) and Statistics Poland, there were about 309,964 horses in Poland. Cold‐blooded horses dominated, constituting about 49% of the population, followed by 36% of warm‐blooded horses and 15% of ponies and small horses. According to PZHK, the quantitative structure of the herd in 2023 was: cold‐blooded—150,720; warm‐blood—101,819; and ponies—57,425.^12^, ^13^

The lack of systematic surveillance of zoonotic agents in the population of horses poses a serious threat to people, especially those employed in direct handling of animals, including veterinarians and zootechnicians. The implementation of a systematic serological monitoring programme in the equine population in Poland is one of the key tools used to reduce the risk of transmission of *Leptospira* infections to humans by estimating the number of horses that are affected or might be exposed.

The aim of the study was to update the data and provide new information regarding the current seroprevalence of leptospirosis in domestic horses in Poland.

## 2 MATERIALS AND METHODS

### 2.1 Sample collection and study area

**FIGURE 1:**
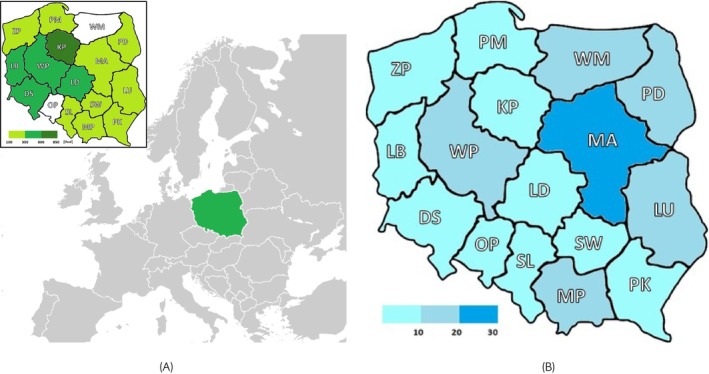
The number of samples collected (A; thsd, thousands), and the average horse population (in thousands) (2019–2023, Statistics Poland) (B) in analysed provinces. Horses in Warmia and Mazury (WM) and Opolskie (OP) provinces were not tested. Provinces: LD, Łódzkie; MP, Małopolskie; MA, Mazowieckie; OP, Opolskie; PK, Podkarpackie; PM, Pomorskie; SL, Śląskie; SW, Świętokrzyskie; WM, Warmia and Mazury; DS, Dolnośląskie; KP, Kujawsko‐Pomorskie; LB, Lubuskie; LU, Lubelskie; PD, Podlaskie; WP, Wielkopolskie; ZP, Zachodniopomorskie.

From January 2019 to December 2023, a total of 4474 serum samples were collected from horses resident in 14 out of 16 provinces in Poland including: Świętokrzyskie (SW), Śląskie (SL), Łódzkie (LD), Zachodniopomorskie (ZP), Wielkopolskie (WP), Pomorskie (PM), Podlaskie (PD), Podkarpackie (PD), Małopolskie (MP), Mazowieckie (MA), Lubuskie (LB), Lubelskie (LU), Kujawsko‐Pomorskie (KP), and Dolnośląskie (DS) (Figure 1A). The samples were collected in cooperation with the State Veterinary Services and Veterinary Inspection throughout the country. The vast majority of the sera obtained came from cold‐blooded horses of the dominant utility type in Poland. The horse population data are presented in Figure 1B.

### 2.2 Microscopic agglutination test

Serum samples were tested by microscopic agglutination test (MAT) using a range of 8 *Leptospira* serovars representing 7 serogroups previously identified serologically, as well as through bacteriological isolation from horses in Europe.^14^, ^15^, ^16^, ^17^, ^18^ The reference strains were provided by the World Organisation for Animal Health (WOAH) Reference Laboratory for Leptospirosis, Amsterdam, the Netherlands (Table 1). The microscopic agglutination test (MAT) is widely considered the gold standard for diagnosing leptospirosis due to its high specificity. However, one of its main limitations is that IgM antibodies—markers of acute infection—typically become detectable only after about 8 days of illness, reaching their peak around the fourth week.^19^ As a result, the sensitivity of the test depends on the stage of infection in an individual animal. Nevertheless, in seroprevalence studies, MAT can provide valuable information about exposure to different *Leptospira* serogroups within an animal population.^1^

**TABLE 1:** *Leptospira* antigens used in the microscopic agglutination test (MAT) assay.

Serogroup	Serovar	Strain
Icterohaemorrhagiae	Icterohaemorrhagiae	RGA
Grippotyphosa	Grippotyphosa	Moskwa V
Sejroe	Sejroe	M 84
Pomona	Pomona	Pomona
Canicola	Canicola	Hond Utrecht IV
Australis	Bratislava	Jez Bratislava
Sejroe	Hardjo	Hardjoprajitno
Javanica	Poi	Poi

Each strain was grown in 10 mL of Ellinghausen–McCullough–Johnson–Harris (EMJH) medium, at 28°C for 6–10 days depending on the serovar. The concentration of bacteria was adjusted to 1–2 × 10^8^ cells/mL using a Helber counting chamber. The sera were initially screened for antibodies to the 12 serovars at a final dilution of 1:100. When agglutination was observed, the relevant sera were end‐point tested using twofold dilutions ranging from 1:100 to 1:25,600. The titre was defined as the highest dilution where ≥50% of the antigen was agglutinated.

The quality control of the MAT was performed by using certified reference *Leptospira* strains and anti‐*Leptospira* rabbit antisera (WOAH Reference Laboratory for Leptospirosis, Amsterdam University Medical Center, Department of Medical Microbiology and Infection Prevention, the Netherlands). Testing of the samples was conducted at the National Reference Laboratory of Leptospirosis, National Veterinary Research Institute in Pulawy, Poland.

### 2.3 Data analyses

All statistical analyses were conducted using Python (version 3.10.12) within the Google Colab environment. Data manipulation and numerical computations were performed using Pandas (version 2.1) and Numpy (version 1.26.4), respectively. Statistical testing was primarily carried out using SciPy (version 1.12.0), while data visualisation was accomplished through Matplotlib (version 3.8.0) and Seaborn (version 0.13.2).

Prevalence was expressed using two complementary measures: absolute prevalence, calculated as the percentage of positive samples relative to the total number of tested samples, and relative prevalence, calculated as the percentage of positive samples for each category (*Leptospira* serogroups and provinces of Poland) among all positive samples.

Pairwise comparisons of prevalence were performed using chi‐squared tests implemented through scipy.stats.chi2_contingency. To account for multiple comparisons, a Bonferroni correction was applied using scipy.stats.false_discovery_control, ensuring a family‐wise error rate below 0.05. To complement the frequentist approach, Bayesian posterior probabilities were estimated using beta distribution sampling (scipy.stats.beta) with parameters *α* = successes + 1, and *β* = failures + 1, thereby quantifying the likelihood that the prevalence of one group exceeded another.

Regional variability in seroprevalence was assessed across 14 provinces using the same statistical framework described above. Proportions of seropositive samples were calculated for each region relative to the total samples tested in that region. Pairwise comparisons between regions were conducted using chi‐squared tests. Additionally, Bayesian posterior probabilities were calculated to evaluate the relative prevalence between regions, incorporating uncertainty into the analysis.

Additionally, Bayesian posterior probabilities were calculated to evaluate the relative prevalence between regions, incorporating uncertainty into the analysis.

Temporal changes in the prevalence of *Leptospira* serogroups over the study period (2019–2023) were assessed by calculating year‐over‐year percentage changes in the proportion of positive results between consecutive years, with 95% Wilson confidence intervals (CIs) for proportions computed using scipy.stats.proportion_confint to quantify uncertainty around each change estimate.

## 3 RESULTS

### 3.1 Overall seroprevalence of *Leptospira* serogroups

Of the 4474 samples of sera collected from horses, 1124 (25.1%) tested positive for antibodies against the pathogenic *Leptospira* serovars included in the eight antigens panel of the routine MAT assay.

**FIGURE 2:**
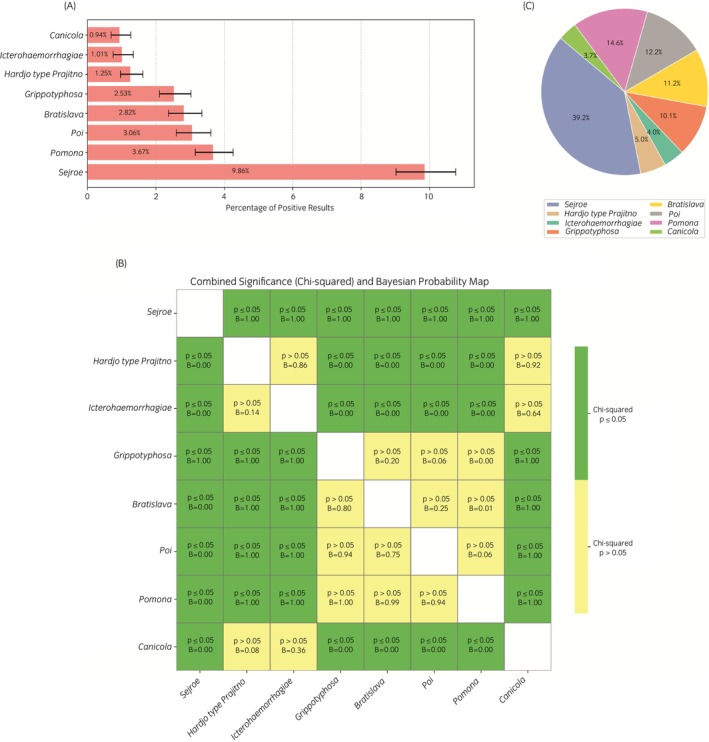
(A) Horizontal bar chart displaying the percentage of seropositive results for each *Leptospira* serogroup, with 95% Wilson confidence intervals represented as error bars. (B) Heatmap visualising pairwise comparisons between *Leptospira* serogroups. Green cells indicate statistically significant differences (*p* ≤ 0.05, chi‐squared test), while yellow cells represent non‐significant differences (*p* > 0.05). Annotations within the cells include the *p* value obtained and Bayesian posterior probability (B). Cells above the diagonal of white squares test whether the positivity rate for the row serogroup exceeds that of the column serogroup; cells below the diagonal of white squares test the converse (column vs. row). Bayesian probabilities indicate the likelihood that the prevalence of the row serogroup exceeds that of the column serogroup in pairwise comparisons. Diagonal cells were excluded from analysis. (C) Pie chart illustrating the relative abundance of each *Leptospira* serogroup.

The seroprevalence of each serogroup, expressed as a percentage of the positive results, ranged from 0.9% for Canicola to 9.9% for Sejroe (see Figure 2A). These differences in prevalence were statistically significant, particularly between Sejroe and less common serogroups such as Canicola, Icterohaemorrhagiae, and Hardjo (*p* ≤ 0.05) (Figure 2B). The Bayesian posterior probabilities further supported these differences in comparisons involving Sejroe, indicating its predominant character with a high confidence (Figure 2B).

The relative frequency of each serogroup, calculated as a proportion of total positive cases, highlighted Sejroe's dominant contribution 441/1124 (39.2%) in the pool of seropositive samples (Figure 2C). Other serogroups detected relatively commonly included Pomona (164/1124; 14.6%), Poi (137/1124; 12.2%), and Bratislava (126/1124; 11.2%). Lower frequencies of seropositive samples were observed for Grippotyphosa (113/1124; 10.1%), Hardjo (56/1124; 5.0%), Icterohaemorrhagiae (45/1124; 4.0%), and Canicola (42/1124; 3.7%).

### 3.2 Regional variability in seroprevalence

**FIGURE 3:**
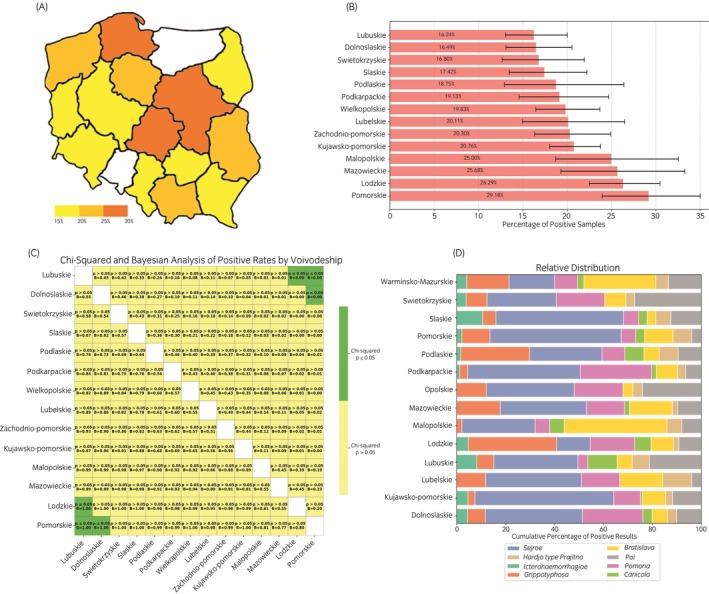
(A) Map of Poland showing the cumulative percentage of positive samples with an initial titre endpoint at 1:100 determined as seropositive for *Leptospira* serogroups across provinces, with colour intensity representing prevalence. (B) Horizontal bar chart displaying the percentage of positive samples by province, with 95% Wilson confidence intervals represented as error bars. (C) Heatmap visualising pairwise comparisons of positive rates between provinces for all *Leptospira* serogroups combined. Green cells indicate statistically significant differences (*p* ≤ 0.05, chi‐squared test), while yellow cells represent non‐significant differences (*p* > 0.05). Annotations within the cells include *p* value thresholds and Bayesian posterior probabilities (‘B’), indicating the likelihood that the positive rate in the row province exceeds that in the column province. (D) Stacked bar chart illustrating the relative distribution of *Leptospira* serogroups within each province, with colours representing different serogroups and bars summing to 100%. For location of provinces see Figure 1.

The analysis of regional seroprevalence across the 14 provinces revealed significant variation in the percentage of positive samples for *Leptospira* serogroups. Of the regions included in the study, Pomorskie recorded the highest percentage of seropositive samples (29.2%), followed closely by Łódzkie (26.3%) and Mazowieckie (25.7%). Conversely, Lubuskie exhibited the lowest prevalence at 16.2%, comparable with Dolnośląskie (16.4%) and Świętokrzyskie (16.8%), also displaying relatively low seropositivity rates (Figure 3A,B).

Statistical analyses of the pairwise differences in the positivity rates between provinces demonstrated significant variation. Pomorskie and Łódzkie were consistently associated with higher positive rates than Lubuskie and Dolnośląskie, with these differences reaching statistical significance (*p* ≤ 0.05) in multiple pairwise comparisons (Figure 3C). Bayesian posterior probabilities (B) further supported these findings, with high probabilities (B = 1.00).

The distribution of individual *Leptospira* serogroups across the provinces also varied significantly. Sejroe was the most dominant serogroup overall, contributing the largest proportion of seropositive results in most regions. In contrast, certain regions showed differing dominance patterns; for example, Łódzkie had a higher relative contribution from Grippotyphosa, while Małopolskie was characterised by an increased prevalence of Australis (Figure 3D). Despite this regional variation, Sejroe maintained its dominant status in most provinces, accounting for a substantial proportion of seropositive samples in the cumulative data.

### 3.3 Serological titration profiles across serogroups

**FIGURE 4:**
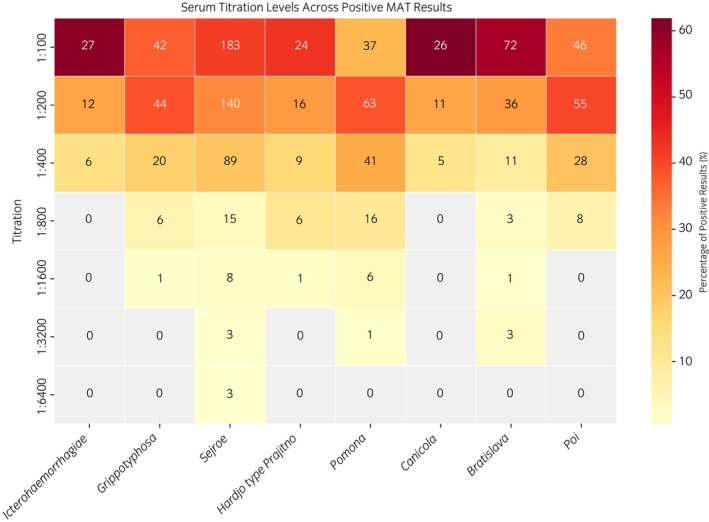
Heatmap showing the distribution of microscopic agglutination test (MAT) titres for *Leptospira* serogroups. Numbers in cells represent the count of seropositive samples at each titre level. The colour intensity corresponds to the percentage of positive samples within each serogroup, with percentages calculated relative to the total positive samples for a given serogroup. Grey cells indicate negative results.

The distribution of serum titration levels varied significantly across *Leptospira* serogroups, highlighting distinct serological patterns (Figure 4). Sejroe demonstrated the highest frequency of positive results, with an initial titre endpoint at 1:100 (183 positive samples), followed by 1:200 (140 positive samples). Seropositivity for Sejroe extended across higher titration levels, including low numbers at 1:3200 and 1:6400.

Bratislava exhibited a distinct profile, with 72 seropositive samples detected at a titre of 1:100 and 36 at 1:200, but few detections at higher titres. In contrast, Pomona had stronger seropositivity at an endpoint titre of 1:200 (63 samples), peaking at this level, with a smaller number of results observed at lower (1:100) and higher (1:400) titres. Similarly, Poi peaked at 1:200 with 55 seropositive samples, while Grippotyphosa showed comparable positivity at 1:200 (44 samples) and 1:100 (42 samples).

Canicola and Icterohaemorrhagiae were among the least frequently detected serogroups, with positivity concentrated at a titre of 1:100 (26 and 27 samples, respectively) and very few positive results beyond 1:200. Hardjo (serogroup Sejroe) displayed an intermediate distribution of titration levels, with 24 positive samples at 1:100, 16 at 1:200, 9 at 1:400, and sporadic detections at 1:800 and 1:1600.

For all serogroups, the number of positive results declined sharply above 1:400 titre, with the exception of Sejroe and Pomona, which maintained sporadic positivity at the highest titration levels.

### 3.4 Temporal trends in *Leptospira* serogroup prevalence

**FIGURE 5:**
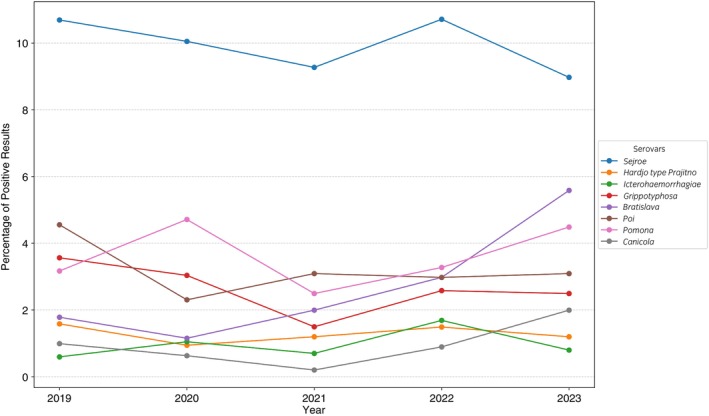
Trends in seroprevalence of *Leptospira* serogroups in equine serum samples in Poland from 2019 to 2023 with an initial titre endpoint at 1:100 determined as seropositive. The number of horse serum samples tested in subsequent years was as follows: 2019 (*n* = 505); 2020 (*n* = 955); 2021 (*n* = 1003); 2022 (*n* = 1008); 2023 (*n* = 1003).

The trends in *Leptospira* serogroups prevalence between 2019 and 2023 showed distinct changes over time for each serogroup, with some exhibiting consistent patterns while others fluctuated significantly (Figure 5, Table 2). Sejroe was the most common serogroup, ranging from 8.9% to 11.0%, with a gradual decline until 2021, a temporary increase in 2022, and a final decline in 2023. Hardjo remained relatively low, fluctuating between 0.9% and 1.6%, showing a clear decline in the first year but slight recovery in the middle period. Icterohaemorrhagiae showed considerable variability, rising to 1.3% in 2020, declining to 0.8% in 2021, peaking at 1.9% in 2022, and dropping to 0.9% in 2023. Grippotyphosa started at 3.5% in 2019, declined steadily to 1.5% by 2021, and rebounded slightly to 2.5% in 2023. Bratislava displayed the clearest upward trend, increasing consistently from 1.7% in 2019 to 5.7% in 2023. Poi experienced a sharp decline from 4.5% in 2019 to 2.3% in 2020, followed by minor fluctuations, stabilising around 3.1% in 2023. Pomona alternated between increases and decreases, ranging between 2.5% and 4.7%. Canicola showed the most dramatic changes, dropping to a low of 0.3% in 2021 before increasing sharply to 2.1% in 2023.

**TABLE 2:** Percent changes in the proportion of seropositive results in individual equine *Leptospira* serovars between consecutive years, expressed as percentages, with 95% Wilson confidence intervals shown in parentheses; initial titre endpoint at 1:100 determined as seropositive.

Serovar	2019–2020		2020–2021		2021–2022		2022–2023	
Sejroe	−6.0	(−9.3, −2.7)	−7.8	(−10.4, −5.1)	15.6	(12.9, 18.2)	−16.3	(−18.9, −13.7)
Hardjo	−40.5	(−41.8, −39.3)	26.9	(26.0, 27.9)	24.4	(23.4, 25.4)	−19.6	(−20.6, −18.6)
Icterohaemorrhagiae	76.3	(75.3, 77.2)	−33.4	(−34.2, −32.5)	141.7	(140.7, 142.6)	−52.7	(−53.7, −51.7)
Grippotyphosa	−14.8	(−16.8, −12.9)	−50.8	(−52.1, −49.4)	72.5	(71.2, 73.7)	−3.4	(−4.7, −2.0)
Bratislava	−35.4	(−36.7, −34.0)	73.1	(72.0, 74.2)	49.3	(47.9, 50.6)	87.6	(85.8, 89.4)
Poi	−49.4	(−51.5, −47.4)	34.2	(32.7, 35.6)	−3.7	(−5.2, −2.2)	3.9	(2.4, 5.4)
Pomona	48.7	(46.7, 50.8)	−47.1	(−48.8, −45.5)	31.4	(29.9, 32.8)	37.0	(35.4, 38.7)
*Canicola*	−36.5	(−37.5, −35.6)	−68.3	(−68.8, −67.7)	347.8	(347.1, 348.4)	123.3	(122.3, 124.4)

## 4 DISCUSSION

Numerous studies conducted in laboratories worldwide have confirmed that leptospirosis is a relatively common infection in horses and can be caused by a broad range of *Leptospira* serovars. Currently available data indicate that the seroprevalence of *Leptospira* infection in horses varies between European countries. Reports indicate a seroprevalence of 6.2%–37.2% in Croatia,^17^, ^20^, ^21^ 1.5%–67.2% in Italy,^22^, ^23^, ^24^ 6.3% in Serbia,^25^ 16.6% in Sweden,^26^ 20.6%–39.0% in Poland,^14^, ^18^, ^27^ and 58.5% in Switzerland, with the most common serogroups being Australis, Autumnalis, Pyrogenes, and Canicola.^28^, ^29^

While all pathogenic leptospires have the theoretical potential to infect horses, in practice only a limited number of serovars are endemic within specific regions or countries. This regional variability reflects the local prevalence of maintenance hosts—typically small rodents—that serve as reservoirs of infection.^1^

The results of our 5‐year study showed a relatively high exposure (25.1%) of horses to *Leptospira* antigens in Poland. Most often, antibodies specific to *Leptospira* serogroup Sejroe (39.2%) were detected in the Polish population of horses. Previous research has reported the isolation of this serogroup from the eyes of horses in Europe.^15^ Three serovars—Hardjo, Sejroe, and Saxkoebing—are commonly found within the Sejroe group in Europe.^30^, ^31^ Since serovar Hardjo has not been documented in Polish cattle, it is unlikely to be the source of the positive reactions we observed.^32^ Instead, our unpublished data suggest that serovar Saxkoebing, which was recently isolated from two field voles (*Microtus agrestis*), may be the likely cause of the widespread serological responses in horses.

Other identified serogroups were Pomona (14.6%), Australis (11.2%), and Grippotyphosa (10.1%). The results were similar to an earlier study carried out on a mixed population of healthy horses (20.6%) in northern Poland.^14^ In contrast to our study, serology testing was performed on only a small number of horses (*n* = 316) with a panel of eight antigens, and indicated that serogroup Grippotyphosa was responsible for most infections. Arent et al. found an almost twice as high prevalence of *Leptospira* spp., reaching 39.0% (*n* = 620). However, a wider range of 17 live antigens representative of the most prevalent serogroups detected in Europe was used in the study.^14^ Similarly to Sobiech and Babicz‐Bury, serogroup Grippotyphosa was also the most common.^27^

Other serogroups against which we detected antibodies at levels higher or equal to 10% were Pomona, Javanica (serovar Poi—12.2%), Grippotyphosa, and Australis, which includes the serovar Bratislava. According to other studies, representatives of the last serogroup Australis may be the only one adapted to horses as natural hosts.^16^, ^33^ Serovar Pomona, type Kennewicki, is recognised as a major animal pathogen in North America. In Europe, however, isolates are more commonly identified as either the Pomona or Mozdok types. The Mozdok serovar has been recovered from horses in Europe^15^, ^16^ and appears more frequently in wild rodents than in livestock. This supports the hypothesis that Mozdok circulates primarily among wildlife populations with occasional transmission to domestic animals.^34^, ^35^

Serogroups against which we detected antibodies at levels greater than 3%, but lower than 10% were: Icterohaemorrhagiae (serovar Icterohaemorrhagiae—3.9%) and Canicola (serovar Canicola—3.7%).

All serogroups mentioned above were previously isolated from horses in Europe,^15^, ^36^, ^37^ and the presence of specific antibodies was also frequently detected in other domestic animals in Poland, which may confirm their endemic occurrence in our country.^38^, ^39^

The latest serological survey from 2021 in Poland estimated the seroprevalence of *Leptospira* species in a population of Polish Arabian horses at 32.2% of serum samples (204 of 615 animals) with antibodies against serogroup Grippotyphosa, the most predominant.^17^ However, the Bratislava serovar (Australis serogroup) was not included in this study, which could have significantly influenced the final result. Considering that our research included a significantly larger number of samples compared to previous studies in Poland (*n* = 4474) and a mixed population of horses in terms of utility type, both probably had a direct impact on the results obtained. This was evident by a change in the serogroups circulating in the population of Polish horses sampled, with the dominant serogroups: Sejroe, Pomona, and Australis. Our study corresponded partially with data from Sweden^19^, ^33^ where Bratislava dominates—16.6% through 49.4% and Grippotyphosa at a low level of 0.4%, and in Italy, where the dominant serogroups were primarily Australis, Canicola, Tarassovi, Icterohaemorrhagiae, Pomona, Sejroe but Grippotyphosa occurred sporadically.^15^, ^16^, ^17^

The results obtained in the present study were most similar to those from Croatia, where the seroprevalence ranged from 6.2% to 23.4% with the most common serogroups being Australis, Pomona, and Grippotyphosa.^17^, ^20^, ^21^ The latest serological research on the prevalence of equine leptospirosis in Croatia in the period from 2012 to 2022 revealed an estimate of 10.8% with the highest seroprevalence found for serogroups Pomona and Grippotyphosa, followed by Sejroe, Icterohaemorrhagiae, and Australis.^20^

Serovars Mozdok (serogroup Pomona) and Grippotyphosa belong to the *Leptospira kirschneri* species. Extensive rodent surveillance in Germany found that 20.7% of captured animals were carriers of *Leptospira*, with 93.1% belonging to the *L. kirschneri* species. Given the geographic proximity, it is likely that similar reservoir dynamics exist in Poland, supporting the role of small mammals as sources of equine infection.^41^

As a zoonosis, *Leptospira* is a pathogen important for public health. In 2022 there were 765 confirmed cases of leptospirosis in humans in the EU/EEA. However, the outbreaks were not always divided into serogroups and/or serovars (ECDC Report).^42^ The most recent detailed information about *Leptospira* occurrence in humans in Poland comes from 2004. The total number of analysed positive samples in patients with clinical symptoms of leptospirosis was only 50. The most common sero0group was Icterohaemorrhagiae (*n* = 27).^3^ Our study in horses was conducted 15 years later in which the most prevalent serogroup was Sejroe. The low number of samples in the Czerwiński and Sadowska‐Todys study and the time gap between the analyses make it impossible to find a link between *Leptospira* outbreaks in horses and humans in Poland.^36^ In another study conducted in Poland, 48 human samples were analysed (2014–2017) and 13 gave positive results (not divided into serogroups/serovars). The material was taken from the patients with clinical symptoms of *Leptospira* spp.^4^ Most of the suspected diagnoses came from the Mazowieckie province, where the horse population is the highest in Poland (Figure 1B) and in which we have detected a high *Leptospira* prevalence in our study (Figure 3). The data received in our trends model (Table 2) indicate that the infectious pressure for leptospirosis in horses, and consequently also for humans, might increase in the following years.

Our research has limitations related to incomplete data related to the breed, utility type, and farming methods of horses that were included. While probabilistic sampling would be ideal, the logistical challenges of random sampling in equine populations necessitated convenience sampling through veterinary clinics and diagnostic laboratories. However, the broad geographic distribution and substantial sample size provide valuable insights into the seroprevalence of leptospirosis in Poland, though extrapolation to the general equine population should be made with appropriate caution. Nevertheless, the utility of a subset of the horses sampled was known, and this largely corresponded to that of the equine population in Poland, which is regularly evaluated by the Statistical Poland.

Our findings reinforce the conclusion that the majority of equine *Leptospira* infections are associated with strains of Mozdok serovar belonging to the *Leptospira kirschneri* species maintained by small rodents in the environment. These results underscore the importance of implementing effective rodent control strategies within stable and farm environments, as well as the need for structured surveillance and prevention programmes for leptospirosis to mitigate risks to both animal and public health. Nonetheless, comprehensive insights into the epidemiology of equine leptospirosis remain limited and will require more extensive research. In particular, it has yet to be determined whether horses can become chronic carriers of pathogenic leptospires—an issue that can only be clarified through renal culture, which was beyond the scope of this study.

## 5 CONCLUSION

This analysis provides valuable data on the circulation of *Leptospira* serogroups across different regions in Poland over a 5‐year period. The high seroprevalence of the zoonotic pathogen *Leptospira* in the Polish horse population underscores the necessity of ongoing monitoring. Continued research will offer essential information to veterinarians and horse breeders, aiding in the protection of individual horses, herds, and people from potential infections.

## FUNDING INFORMATION

This study was funded by the Multiannual Program 2019–2023 ‘Protection of animal health and public health’ under task number 11 ‘Assessment of the epidemiological situation of leptospirosis in pigs, horses, cattle and small ruminants (W311)’.

## CONFLICT OF INTEREST STATEMENT

The authors declare no conflicts of interest.

## AUTHOR CONTRIBUTIONS

**Jacek Żmudzki:** Writing – review and editing; conceptualization. **Monika Ostrowska:** Writing – review and editing; visualization; formal analysis. **Zbigniew Arent:** Writing – review and editing; visualization; formal analysis. **Maciej Frant:** Investigation; validation; software. **Maciej Kochanowski:** Methodology; software. **Agnieszka Nowak:** Software; methodology; formal analysis. **Sylwia Zębek:** Writing – review and editing; formal analysis. **Damian Kalinowski:** Conceptualization; writing – original draft; formal analysis; project administration; supervision; investigation. **Katarzyna Podgórska:** Methodology; data curation; software; writing – review and editing; investigation; resources.

## DATA INTEGRITY STATEMENT

Jacek Żmudzki and Monika Ostrowska had full access to all the data in the study and take responsibility for the integrity of the data and the accuracy of the data analysis.

## ETHICAL ANIMAL RESEARCH

Research ethics committee oversight not currently required by this journal: The study was performed on material obtained from a National serological monitoring programme.

## INFORMED CONSENT

Not stated.

## Data Availability

The data that support the findings of this study are available upon reasonable request from the corresponding author. Open data sharing exemption granted by the editor.
